# Surgical Site Bacterial Infection in a General Hospital, Al-Jouf Region, Saudi Arabia: A Retrospective Study

**DOI:** 10.7759/cureus.43613

**Published:** 2023-08-17

**Authors:** Suliman Al-Khalidi, Amany A Ghazy, Ashraf A Taha, Norah Bassam Fahad Alrasheid, May Hamad Saad Al-Qaed, Norah Sattam Homod Alrwuili, Asayel Mojidea Mahdi Alshammri, Amlak Salman Ali Almatrafi, Reham Thani Mudasher Al-Ruwaili, Amirah Mojidea Mahdi Alshammari

**Affiliations:** 1 Department of Urological Surgery, Prince Metab Hospital, Ministry of Health, Sakaka, SAU; 2 College of Medicine, Jouf University, Sakaka, SAU; 3 Department of Internal Medicine, Prince Metab Hospital, Ministry of Health, Sakaka, SAU

**Keywords:** post-operative problems, kingdom of saudi arabia (ksa), al-jouf region, prevalence, surgical site infection (ssi)

## Abstract

Background

Surgical site infection (SSI) is a major healthcare problem with a great impact on patient morbidity, mortality, and healthcare cost all over the world. It accounts for 20% of healthcare-associated infections (HAIs), with higher frequency in low- and middle-income countries where it affects about 30% of the patients undergoing surgery.

Aim

The current study aims to assess the prevalence of SSI in a general hospital in Sakaka, Al-Jouf region, Saudi Arabia. The types of bacteria causing SSI were also determined.

Subjects and methods

A retrospective cross-sectional study was done by reviewing the hospital records of patients who got SSI during the period between 2020 and 2022. Data collection was done during 2022 and 2023 after taking ethical approval and permission from the hospital management.

Results

The number of patients who underwent surgical procedures during 2020, 2021, and 2022 were 689, 867, and 1119, respectively. Most of the cases were cholecystectomy and appendectomy. The cases that developed surgical site infection after cholecystectomy and/or appendectomy during 2021 and 2022 were 15.45% and 9.29% cases, respectively, and they were mainly associated with appendectomy. A culture and sensitivity test revealed *methicillin-resistant Staphylococcus aureus (MRSA)* and *Klebsiella pneumonia*. Nearly all patients have received ciprofloxacin for seven days and improved with treatment.

Conclusion

The number of cases that developed SSI has decreased gradually due to the application of infection control measures and strict follow-up.

## Introduction

A surgical site infection (SSI) means an infection of a surgical wound within 30 days, or up to one year with the use of an implantable device, after a surgical procedure. Globally, it is the third most common nosocomial infection [1].

This healthcare problem has a great impact on patient morbidity, mortality, and healthcare costs all over the world. It accounts for 20% of healthcare-associated infections (HAIs), with a higher frequency in low- and middle-income countries where it affects about 30% of the patients undergoing surgery [2-4]. SSIs have a significant additional cost burden [5].

Prevention and control of SSI is a key constituent of patient safety and quality care. It is important to determine the common pathogens causing SSI and improve the knowledge of healthcare workers about these pathogens and how to prevent them to ensure better-wound care and patient safety [6].

In Saudi Arabia, there is a very limited number of recent studies describing the prevalence of SSI. Despite the use of updated approaches in disinfection and treatment, there is the emergence of resistant infections that may affect the postoperative prognosis. In addition, there is a lack of follow-up data to know the precise percentage of SSI in each hospital, the kinds of operations followed up, and the types of organisms, and to ensure the proper application of infection control measures. Thus, the current study aims to assess the prevalence of SSIs in a general hospital in Sakaka, Al-Jouf region, Saudi Arabia. The types of bacteria causing SSI were also determined. This will help in determining the type of infection, and new infection control policies or protocols can be designed for each hospital.

## Materials and methods

Study design

This was a retrospective cross-sectional study during the period from January 2020 to December 2022. The hospital records of 2,675 patients who underwent surgical procedures during 2020, 2021, and 2022 were reviewed to determine the number of patients who developed SSI.

Study duration

The study was completed in six months. The collection of data was done through 2022/2023.

Sampling technique

After taking ethical approval and permission from the hospital management, we accessed infection control files to determine the types of surgical procedures followed, the number of cases with SSI, the treatment used, and the response to treatment.

Inclusion criteria

Any patient who underwent a surgical operation from January 2020 to December 2022, a resident of the Al-Jouf region, was included in the study.

Exclusion criteria

Any patient with chronic, autoimmune, and/ or immune deficiency diseases was excluded from this study.

Ethical approval

The research proposal was approved by the Local Committee of Bioethics (LCBE), Jouf University, on 19-4-2022 (approval number: 4-08-43).

Data analysis procedure

Data were fed to the computer and analyzed using IBM SPSS software package version 20.0. (Armonk, NY: IBM Corp). Qualitative data were described using numbers and percentages.

## Results

**Figure 1:**
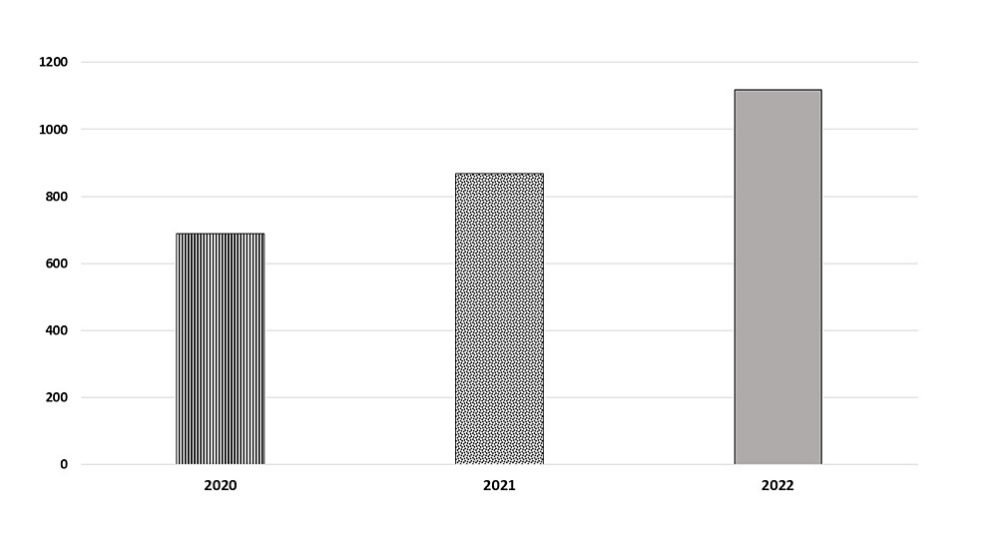
Number of general surgical procedures during the period between 2020 and 2022

The numbers of patients who underwent surgical procedures during 2020, 2021, and 2022 were 689, 867, and 1119, respectively (Figure 1).

Subject demographic data

The current study included 2675 with ages between 24 and 59 years (34±7.5 years). There were 54.9% males and 55.1% females.

Surgical procedures

It was noticed that the number of surgical operations during 2020 was very low due to the coronavirus disease 2019 (COVID-19) pandemic, as many cases were transferred to King Abdulaziz Specialized Hospital. There was a gradual increase in the number of surgical operations later.

**Figure 2:**
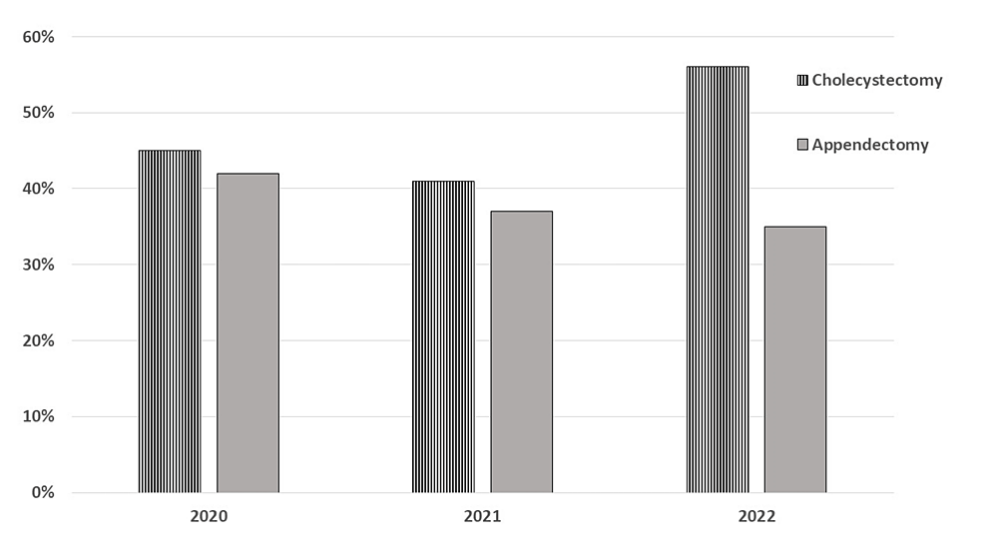
Number of surgical procedures during the period between 2020-2022

It was noticed that most of the cases were cholecystectomy and appendectomy. Cholecystectomy represents 45.3%, 40.8%, and 55.7% of surgical procedures during 2020, 2021, and 2022, respectively, while appendectomy represents 42%, 36.6%, and 34.8% of surgical procedures during 2020, 2021, and 2022, respectively (Table 1 and Figure 2).

**Table 1:** Number of surgical procedures during the period between 2020 and 2022

Year	Cholecystectomy			Appendectomy		Total number of surgical procedures
	Number	%	Number		%	
2020	312	45.3%	289		42%	689
2021	354	40.8%	317		36.6%	867
2022	623	55.7%	389		34.8%	1119

Cases that developed surgical site infection

Regarding the number of cases that developed surgical site infection after cholecystectomy and/or appendectomy, the results are summarized in Table 2. For 2020, we could reach all files of patients who developed SSI. During 2021 and 2022, the numbers of SSIs were 134 (15.45% of the surgical procedures) and 104 cases (9.29% of the surgical procedures), respectively (Table 2). There was a gradual decrease in the number of SSI cases from 2021 to 2022. These cases were mainly associated with appendectomy, which is considered a dirty wound with a high risk of contamination, particularly if a perforated appendix. A culture and sensitivity test was done and the organisms isolated were *methicillin-resistant Staphylococcus aureus (MRSA)* and *Klebsiella pneumonia*. Nearly all patients had received ciprofloxacin for seven days and improved with treatment.

**Table 2:** Cases with surgical site infections

Year	Surgical site infections	
	Number	%
2020	NA	NA
2021	134	15.45%
2022	104	9.29%

## Discussion

There are many contributors to the occurrence of SSI during the preoperative, intraoperative, or postoperative periods [7]. Until the introduction of antiseptic surgery in the nineteenth century, most surgical wounds became infected and resulted in a high mortality rate [8]. Then, developments in the study of microbiology have made surgery safer. However, the overall incidence and prevalence of SSI remain high and the postoperative complications have a significant impact on morbidity and mortality [1,9].

Prevention and control of SSI is a key constituent of patient safety and quality care. It is important to determine the common pathogens causing SSI and improve the knowledge of healthcare workers about these pathogens and how to prevent them in order to ensure better wound care and patient safety [6]. Thus, the current study aimed to assess the prevalence of surgical site infection in a general hospital in Sakaka, Al-Jouf region, Saudi Arabia. Also, the types of bacteria causing SSI were determined.

Results clarified that most of the surgical procedures during this period were cholecystectomy and appendectomy. Cholecystectomy represents 45.3%, 40.8%, and 55.7% of surgical procedures during 2020, 2021, and 2022, respectively, while appendectomy represents 42%, 36.6%, and 34.8% of surgical procedures during 2020, 2021, and 2022, respectively.

It is known that wounds of cholecystectomy and appendectomy may be semi-clean and/or dirty with a high risk of contamination. Regarding the number of cases who developed surgical site infection during 2020, we cannot reach all files for patients who develop SSI. During 2021 and 2022, the numbers of patients who get SSIs were 134 (15.45%) and 104 (9.29%), respectively. There was a gradual decrease in the number of SSI cases from 2021 to 2022. These cases were mainly associated with appendectomy, which is considered a dirty wound with a high risk of contamination, particularly for a perforated appendix.

This is in partial agreement with a study done at King Abdulaziz University Hospital (KAUH), between 2013 and 2017, to assess post-appendectomy SSI and its predisposing factors. They found that 7.2% of post-appendectomy patients have developed SSI. They noticed that SSI was related more to open surgery, perforated appendicitis, longer duration of operation, and/or hospital stay [7].

A study conducted in a tertiary-care hospital in Ho Chi Minh City, Vietnam, reported that the prevalence of SSI among surgical patients was 14.3%. Researchers noticed that trauma and dirty wounds were the common risk factors associated with the occurrence of SSI. They concluded that the prevalence and antimicrobial use of SSI among surgical patients is inconsistent with the declared guidelines, and pathogens were resistant to commonly used antimicrobials [10]. Another study in a tertiary care setting in Mumbai, India, showed that the SSI rate in general surgery and ICU departments between June 2011 and March 2013 was 11%. Many risk factors were determined as age (>55 years), uncontrolled diabetes mellitus, immune-suppressed patients, emergency cases, dirty wounds, cholecystectomy, multilayer closure of the wound, prolonged duration of the surgery, and/or hospital stay [11].

Alkaaki et al., (2019) reported that SSI after open abdominal surgery versus laparoscopic operations was 35% versus 4% [12]. They noticed that the open approach, emergency operation, and longer procedures are potential risk factors for SSI and recommend tailoring the prophylactic regimen used according to the commonly isolated organisms. Moreover, SSI after some surgeries, such as spinal surgery, varies greatly between 0.5% and 18%. This may be linked to longer procedure durations and/or postoperative hospital stays, hypertension, and blood transfusion [13,14].

A culture and sensitivity test was done and the organisms isolated were MRSA and *Klebsiella pneumonia*. Nearly all patients received ciprofloxacin for seven days and improved with treatment. This is in agreement with a study conducted in King Fahd University Hospital, Al Khobar, Saudi Arabia. They assessed the SSI prevalence in orthopedic practice, identified the risk factors, and determined the causative agents between January 2006 and December 2011. They found that 2.55% of patients developed SSI and the commonest infective organism was MRSA followed by Acinetobacter species, Pseudomonas species, and Enterococcus species [15]. Furthermore, Akhter et al., (2016) reported the highest rates of *Staphylococcus aureus*, *Escherichia coli*, and *Klebsiella spp* as causative organisms for SSIs [11].

In Saudi Arabia, Rawabdeh et al. (2016) investigated the possible predictors for SSIs in a teaching hospital [16]. They followed the patients with surgical wounds from admission until discharge. They found that *Staphylococcus aureus* and *Escherichia coli* were the main causative organisms of SSI. The incidence rate of surgical site infections was 11.4% [16]. Furthermore, Koumu et al., (2021) investigated all patients who underwent appendectomy at King Abdulaziz University Hospital (KAUH) between 2013 and 2017 [7]. They noticed that 31 patients out of 433 developed SSI post-appendectomy. They reported that perforated appendicitis, open surgical technique, and longer duration of the operation were the most frequent causes of SSI [7].

Contrary to our results, a Saudi surveillance study was performed in four hospitals of the Ministry of National Guard Health Affairs between 2007 and 2016. They noticed that more than 60% of SSIs are caused by multidrug-resistant (MDR) Acinetobacter, Klebsiella, and Escherichia coli spp. In addition, MRSA was less frequent. They concluded that the presence of these resistant pathogens will augment the clinical and economic impacts of SSI [4].

The main limitations of the current study were that some data were missed during the year 2020 from the records in the infection control unit. Also, we could not correlate patients’ demographic data or medical conditions with if they had chronic diseases or not with the treatment.

## Conclusions

Cholecystectomy and appendectomy have the highest rates of surgical procedures in the hospital and the number of cases that developed surgical site infections has decreased markedly from 2020 to 2022. This may be due to the application of infection control measures (IPC bundle) and strict follow-up.
